# Exosome engineering for targeted therapy of brain-infecting pathogens: molecular tools, delivery platforms, and translational advances

**DOI:** 10.3389/fmedt.2025.1655471

**Published:** 2025-10-02

**Authors:** Hope Onohuean, Sarad Pawar Naik Bukke, Chandrashekar Thalluri, Kasim Sakran Abass, Yahya Essop Choonara

**Affiliations:** ^1^ Wits Advanced Drug Delivery Platform Research Unit, Department of Pharmacy and Pharmacology, School of Therapeutic Sciences, Faculty of Health Sciences, University of the Witwatersrand, Johannesburg, South Africa; ^2^ Biomolecules, Metagenomics, Endocrine and Tropical Disease Research Group (BMETDREG), Kampala International University, Western Campus, Ishaka-Bushenyi, Uganda; ^3^ Department of Pharmaceutics and Pharmaceutical Technology, Kampala International University, Western Campus, Uganda, East Africa; ^4^ Faculty of Pharmaceutical Science, Assam Down Town University (AdtU), Guwahati, Assam, India; ^5^ Department of Physiology, Biochemistry, and Pharmacology, College of Veterinary Medicine, University of Kirkuk, Kirkuk, Iraq; ^6^ Wits Infectious Diseases and Oncology Research Institute, Faculty of Health Sciences, University of the Witwatersrand, Johannesburg, South Africa

**Keywords:** exosome, engineering, blood-brain barrier (BBB), central nervous system (CNS), infections, targeted drug delivery

## Abstract

Central nervous system (CNS) infections caused by pathogens such as HIV, Herpes simplex virus, Cryptococcus neoformans, and Toxoplasma gondii remain among the most difficult to treat due to the physiological barrier posed by the blood-brain barrier (BBB), pathogen latency, and systemic toxicity associated with conventional therapies. Exosome-based delivery systems are becoming a game-changing platform that can solve these therapeutic problems using their natural biocompatibility, minimal immunogenicity, and capacity to cross the BBB. This review current developments in exosome engineering that aim to make brain-targeted therapy for neuroinfectious illnesses more selective and effective. Much focus is on new molecular methods like pathogen-specific ligand display, aptamer conjugation, lipid modification, and click–chemistry–based surface functionalisation. These methods make it possible to target diseased areas of the brain precisely. Exosomes can also carry therapeutic payloads, such as anti-viral and antifungal drugs, gene editing tools like CRISPR/Cas9 and siRNA, and more. This makes them helpful in changing pathogens' persistence and the host's immunological responses. The paper tackle problems with translation, such as biodistribution, immunogenicity, GMP production, and regulatory issues. Future possibilities like synthetic exosomes, combinatory medicines, and delivery design that uses AI. The combination of nanotechnology, molecular biology, and infectious disease therapies shows that exosome engineering offers a new way to meet the clinical needs that are not satisfied in treating CNS infections.

## Highlights

• Exosomes possess an inherent ability to cross the blood-brain barrier (BBB), offering a promising platform for CNS-targeted drug delivery.
• Surface engineering of exosomes with ligands (e.g., RVG, transferrin, mannose, aptamers) enables selective targeting of infected brain cells (e.g., microglia in HIV, neurons in toxoplasmosis, or meninges in cryptococcosis).
• Engineered exosomes have therapeutic success in HIV encephalitis, cryptococcal meningitis, cerebral toxoplasmosis, and tuberculous meningitis models.
• Intranasal, intravenous, intrathecal, and intraventricular administration are discussed to optimise CNS biodistribution and treatment efficacy.

## 1 Introduction

Infections of the central nervous system (CNS) represent a clinically and therapeutically challenging group of infections caused by multiple pathogens, including Human Immunodeficiency Virus (HIV), Herpes Simplex Virus (HSV), Cryptococcus neoformans, and Toxoplasma gondii. Such infections can lead to severe neurological issues, neuroinflammation, and in some cases, encephalitis or meningitis that can be fatal, particularly in the immunocompromised population (1, 2).

The blood-brain barrier (BBB) is among the greatest obstacles in treating CNS infections. It is a selective physiological barrier which prevents the entry of most drugs into the brain (3). While it protects the CNS from potential hazards due to toxic substances, it is also the barrier that limits medications to areas of the brain parenchyma needed for addressing an infection. Additionally, many cases of standard treatment fail to target the CNS effectively, are damaging to the rest of the body, and are non-targeted against certain pathogens, which has an overall negative impact reducing efficacy and increasing any risk of pathogens persisting or recurring in the body (4, 5). Exosomes—extracellular vesicles (30–150 nm) secreted by nearly all cell types—are currently being heavily researched as an option for targeted drug delivery to the brain (6). They are naturally suitable for crossing the blood brain barrier (BBB), transporting biological cargo (e.g., proteins, nucleic acids, and lipids), effectively interacting and regulating with the recipient cell (7). The research examining their natural biological role in intercellular communication, low immunogenic profile, and modifiable surface properties, leads us to consider them as delivery vehicles for agents specifically targeting infected CNS sites (8). This review will examine the cutting-edge developments in exosome engineering for infectious pathogens of the brain and will consider the most recent developments in regard to surface modifications, pathogen-targeted delivery using ligand displays, and molecular payloads including CRISPR/Cas9 and siRNA (9). This will also include engineered delivery of anti-viral, anti-fungal, and neuro-protective drugs by modified exosomes (10). It will also consider the difficulty of making these drugs deliver to the target correctly in application; i.e., immunogenicity, biodistribution, clinical scale-up, and regulatory considerations. The field of nanobiotech and it's potential when combined with the treatment of infectious diseases is relatively novel and potentially revolutionary in providing a sophisticated and considerate mechanism to combat CNS infections more precisely, accurately and efficiently (11).

## 2 CNS-Infecting pathogens, pathogenesis and therapeutic barriers

Infections of the central nervous system (CNS) caused by a variety of microorganisms or pathogens still create a large global burden of diseases, that especially affects immune compromised individuals, like those with advanced HIV/AIDS, tuberculosis (TB), etc. (12, 13). Due to CNS infections, the microorganism or pathogen inflicts damage to the nervous system, both acutely and chronically, increasing the difficulty of therapy, due to the fact that it can occupy CNS space, avoid the host immune system, and develop powerful resistance to drugs (14, 179). Among the major pathogens of the brain are;

### 2.1 Human immunodeficiency virus (HIV)

HIV invades the CNS much sooner in the course of the infection, and establishes latent reserves within microglial cells and perivascular macrophages (15). The virus can cause HIV-associated neurocognitive disorders (HAND), characterized by memory loss, motor impairment, and behavioral changes (16). These effects generally emerge from chronic neuroinflammation, viral neurotoxiins, and even neuronal damage following immune activation-not simple infection of neurons (17).

### 2.2 Herpes simplex virus (HSV-1 and HSV-2)

Herpes simplex virus (HSV) is sometimes associated with herpes simplex encephalitis (HSE), which can be serious and potentially life threatening without treatment (18). After the first infection, the virus may remain dormant in the trigeminal or sacral ganglia which may reactivate causing additional episodes and potentially spreading into the brain. While either the HSV-1 or HSV-2 subtypes can cause HSE, HSV-1 is the most common cause of sporadic viral encephalitis in adults. Its rapid timing, propensity for causing temporal lobe necrosis, and long-term cognitive impairment are notable (19, 20).

### 2.3 Cryptococcus neoformans

This encapsulated fungal infection is one of the leading causes of fungal meningitis, especially in people with weak immune systems, like those with AIDS or who have had a transplant (21). Cryptococcus spreads through the blood and gets into the CNS. It then uses “Trojan horse” mechanisms (within infected phagocytes) to get over the BBB. It can live and reproduce in the subarachnoid space. Cryptococcal meningitis, which is what happens when this happens, is marked by increased intracranial pressure, changes in mental status, and a high death rate (22).

### 2.4 Toxoplasma gondii

T. gondii is a common protozoan that lives inside cells and causes toxoplasmic encephalitis (TE), especially in people with weak immune systems (23). The parasite causes a long-lasting, hidden infection in the central nervous system (CNS), mainly in neurons and astrocytes. When latent tissue cysts in the brain become active again, they can cause seizures, localised neurological impairments, and encephalopathy (24, 25).

## 3 Therapeutic barriers and challenges in the management of brain infections

### 3.1 Poor blood-brain barrier (BBB) permeability

The BBB is a very selective and well-controlled barrier that keeps most drugs from getting into the brain. Many therapeutic compounds, including big hydrophilic medicines, cannot get through the BBB in sufficient concentrations, leading to subtherapeutic levels at the site of infection (26).

### 3.2 Systemic toxicity and lack of targeting specificity

Drugs often need to be given at large systemic doses to penetrate the CNS effectively, which raises the risk of off-target effects and toxicity. This is especially bad with antifungals like amphotericin B or antiretroviral medicines, which can hurt the kidneys or liver (8).

### 3.3 Pathogen persistence and latency

CNS pathogens often evade immune surveillance and persist in latent or quiescent forms within protected niches such as microglia (HIV), neuronal ganglia (HSV), or neurons (*T. gondii*) (27). These reservoirs are inaccessible primarily to conventional drugs and can serve as sources of reactivation, contributing to recurrent or chronic disease (28).

### 3.4 Neuroinflammation and immune-mediated damage

In many CNS infections, neuronal damage results from direct pathogen activity and an exaggerated immune response. This neuroinflammatory milieu makes treatment much harder by damaging the BBB, changing how drugs are transported, and making tissue damage worse. Because of these problems, we need new, focused delivery methods to cross the BBB quickly, target just sick cells, and deliver therapeutic payloads with as few side effects as possible. Exosome-based drug delivery systems have much potential to get around these problems (28).

Importantly, the biologic behavior of each CNS pathogen will greatly impact the nature of exosome-based therapies. For example, HIV establishes latency in microglia and macrophages, and designed exosomes with ligands (e.g., RVG peptide or anti-gp120 antibodies) to deliver siRNA or CRISPR cargo can be used to target these reservoirs. HSV, on the other hand, persists in neuronal ganglia and reactivates in episodic fashion; exosomes containing anti-ICP0 siRNA or decorated with gD-binding peptides can provide pathogen-specific suppression (29). Cryptococcus crosses the BBB by “Trojan horse” mechanisms and exists in the meninges, which endorses the use of mannose- or dectin-1-modified exosomes packaged with amphotericin B or fluconazole. Finally, Toxoplasma gondii forms latent cysts in neurons and astrocytes; thus, exosomes that contain aptamers to SAG1 or CD36 in combination with a CRISPR or siRNA cargo has an added therapeutic value. By connecting pathogen biology with the design of exosomes, we can rationally dictate how to optimize therapies to manage barriers associated with latency, intracellular sanctuary and immune evasion (30).

## 4 Exosomes: biology, biogenesis, and relevance in CNS therapy

Exosomes are tiny extracellular vesicles of nano-sized (30–150 nm in diameter) that are very important for cell-to-cell communication because they carry a wide range of biological components, such as proteins, lipids, DNA, mRNA, and non-coding RNAs like microRNAs (miRNAs) (31). They come from the endosomal compartment and are discharged into the outside world when multivesicular bodies (MVBs) fuse with the plasma membrane (32).

### 4.1 Biogenesis of exosomes

**Figure 1:**
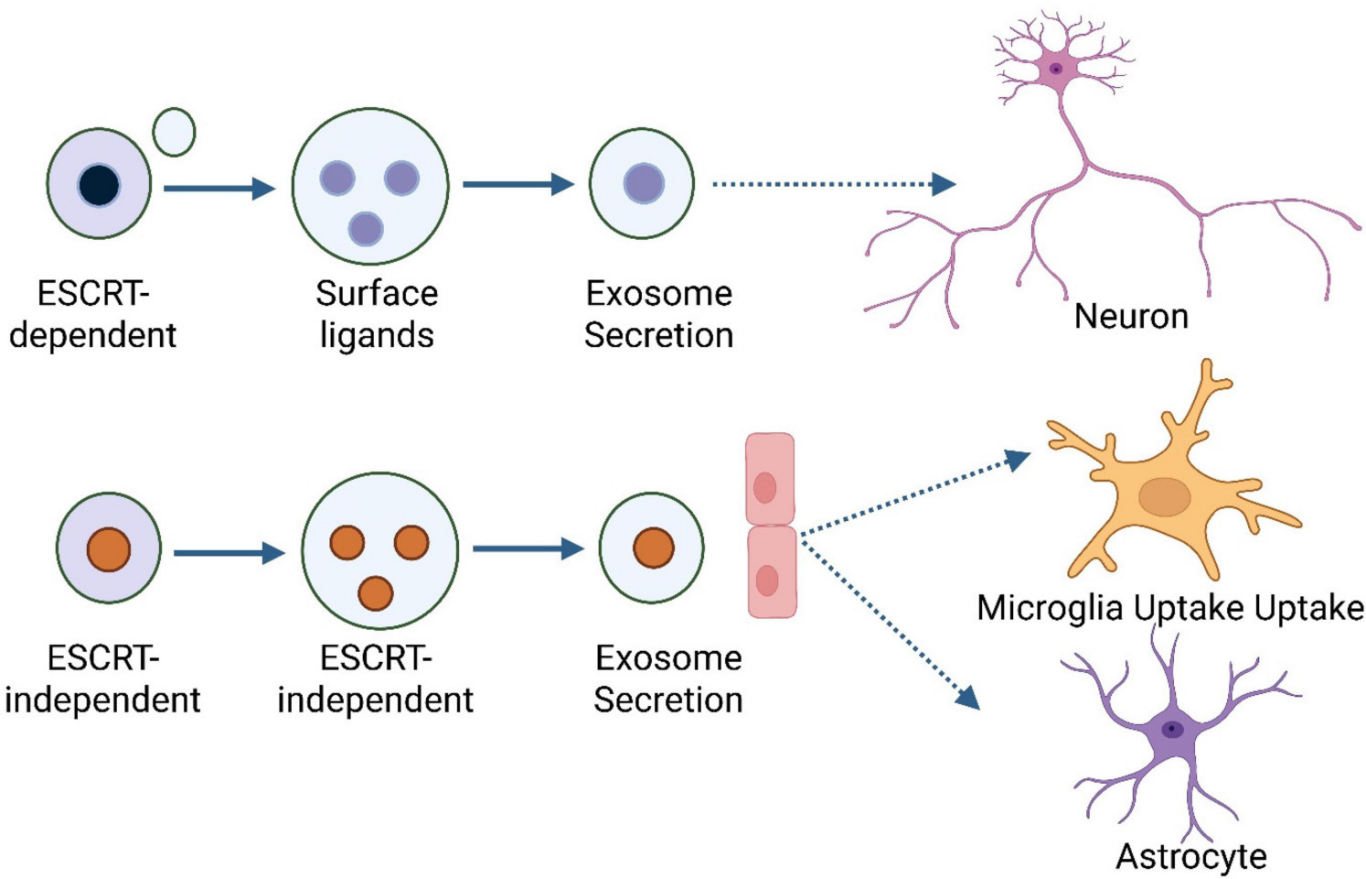
Biogenesis and natural function of exosomes.

The formation of exosomes is a tightly regulated process involving both ESCRT-dependent and ESCRT-independent ways that exosomes develop, and the process is strictly controlled (33). The Endosomal Sorting Complex Required for Transport (ESCRT) machinery helps the endosomal membrane bud inward to make intraluminal vesicles (ILVs) inside MVBs in the ESCRT-dependent pathway. ESCRT-0, -I, -II, and -III are important parts of ESCRT that help choose cargo, change the shape of membranes, and split vesicles (34). Proteins like tetraspanins (CD63 and CD81) and lipids like ceramides help make ILVs by bending membranes and sorting specific cargo into exosomes. These pathways do not depend on ESCRT Figure 1. When MVBs are fully grown or mature, they can either fuse with lysosomes for degradation or with the plasma membrane to release their ILVs as exosomes into the extracellular space (35).

### 4.2 Molecular composition and functions

Exosomes carry unique surface markers, including CD9, CD63, CD81, ALIX, and TSG101. They also have cargo important for their function, such as miRNAs controlling gene expression in cells that take them in (36). Proteins and enzymes change how the immune system works or assist cells in metabolising, as well as lipids that help keep membranes stable and make it easier for vesicles to fuse. These molecular contents are carefully packaged, often showing the health or illness condition of the parent cell. This makes exosomes possible indicators for CNS ailments (37). Comparison with other delivery systems, exosomes have advantages as CNS delivery vehicles including but not limited to their ability to cross the blood–brain barrier (BBB), low immunogenicity, and inherent biocompatibility (38). Conversely, liposomes are established nanocarriers with sufficient drug loading capabilities and scalable manufacture; however, they are often labile to rapid clearance and have limited BBB penetrability. Polymeric nanoparticles have tunable physicochemical properties and controlled release, but may present cytotoxicity and an immune response, which negatively impacts long-term safety (39). Viral vectors are highly efficacious in gene delivery but raise fears surrounding immunogenicity, mutagenesis, and communication among regulatory hurdles. Exosomes, in comparison, afford a natural targeting mechanism and decreased toxicity (40). Nevertheless, challenges remain for exosomes in the form of scalability, heterogeneity, and reliable data for standardization. Hence, there is no single system which is the universally optimal solution, but exosomes can balance efficacy with safety for therapies in CNS infections (as long as current limitations are solved by future investigations).

### 4.3 Exosomes in CNS communication and therapy

Neurones, astrocytes, oligodendrocytes, and microglia are all neural cells that release exosomes in the CNS. Moving bioactive molecules between cells helps with important bodily functions such as synaptic plasticity, neuronal survival, myelination, and neuroinflammation (41). Their intrinsic capacity to breach the BBB and tendency to go towards inflamed or infected neural regions make them suitable delivery vehicles for therapeutic interventions in CNS diseases. Recent studies have shown that exosomes can be modified to carry therapeutic substances (including antiretrovirals, antifungal medicines, and CRISPR/siRNA constructs) to parts of the brain that are infected or injured (9). Their low immunogenicity, long circulation half-life, and capacity to avoid phagocytic clearance make them even better candidates for brain-targeted therapy, especially in CNS infections when standard treatments do not reach therapeutic levels (9).

## 5 Molecular engineering of exosomes for brain pathogen-specific targeting

**Figure 2:**
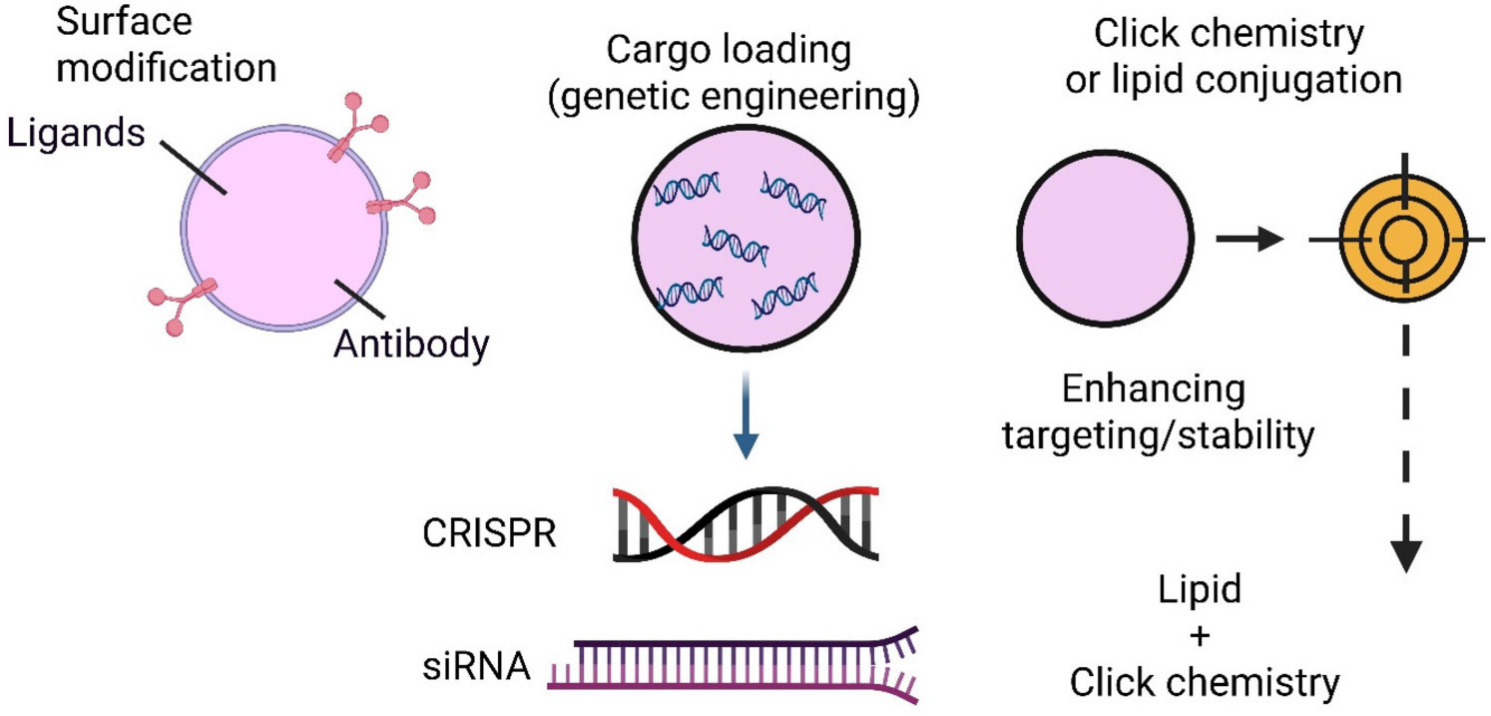
Strategies for molecular engineering of exosomes.

Developing engineered exosomes as nanocarriers for targeted therapy of CNS infections has emerged as a cutting-edge approach in nanomedicine (42). Native exosomes exhibit some ability to cross the blood-brain barrier (BBB) and home to specific tissues, but molecular engineering significantly enhances their precision, cargo-loading capacity, and disease-specific targeting (43). This section explores the diverse surface modification techniques, genetic engineering tools, and chemical conjugation strategies for optimising exosomes for pathogen-specific CNS therapy (44) (Figure 2).

### 5.1 Exosome surface modification strategies for targeting CNS infectious

Surface engineering is a central strategy in exosome modification, enabling specific interaction with infected cells or facilitating BBB penetration. Two primary approaches are employed: ligand display and membrane anchoring of targeting moieties (45). One of the most important parts of exosome-based targeted therapy is ensuring that the therapeutic vesicles are sent directly to the cells in the CNS infected with pathogens. Pathogen-specific ligand display is a method that makes this level of accuracy possible. It involves targeting moieties (ligands) on the surface of exosomes so that they can find and bind to pathogen-related indicators or host cell receptors that have been changed by infection. This method makes exosomes more selective, easier to take up, and more effective as treatments, especially when it comes to diseases that infect the brain, such as *Mycobacterium tuberculosis*, *HIV*, *Cryptococcus neoformans*, *Herpes simplex virus* (HSV), and *Toxoplasma gondii* (44) Table 1. The principles of pathogen-specific ligand display involves engineering the exosome membrane to present molecular recognition elements that bind selectively to: Pathogen-specific antigens (e.g., viral glycoproteins, fungal capsular components). Host cell receptors are regulated during infection (e.g., mannose receptor CD206, ICAM-1) and Cellular stress or inflammatory markers in infected brain tissue. These ligands may include: Peptides, Antibodies or antibody fragments, Aptamers, Lectins or sugar moieties and single-chain variable fragments (scFvs) (51) Table 1.

**Table 1:** Ligands for specific brain-infecting pathogens.

Pathogen	Target marker	Ligand used	Delivery strategy
HIV	gp120, infected microglia	Anti-gp120 scFv, RVG peptide	Lamp2b fusion, antibody conjugation (46)
Toxoplasma gondii	SAG1, CD36	SAG1-specific aptamer	Genetic fusion or aptamer-lipid insertion (47)
Cryptococcus neoformans	Glucuronoxylomannan (GXM)	Mannose, Dectin-1 peptide	Lipid-inserted DSPE-PEG-mannose (48)
Mycobacterium tuberculosis (CNS-TB)	Mannose receptor (CD206), ICAM-1	Mannose, anti-CD206 antibody	Lipid insertion, chemical conjugation (49)
Herpes simplex virus (HSV)	gD/gB glycoproteins	gD-binding peptide, anti-gB antibody	Genetic display or covalent conjugation (50)

#### 5.1.1 Engineering Strategies for Ligand Display

##### 5.1.1.1 Genetic Fusion to Exosomal Membrane Proteins

The most common and stable method involves genetically fusing a targeting ligand to an exosome-enriched membrane protein like Lamp2b (Lysosomal-associated membrane protein 2b), CD63, CD9, CD81 (tetraspanins) and PDGFR or VSVG (viral glycoproteins) (52). For instance, the Lamp2b fused with the RVG peptide allows the exosomes to bind to acetylcholine receptors on neuronal cells, widely applied for targeting CNS-infecting viruses such as rabies and HIV (53) Table 1.

##### 5.1.1.2 Chemical Conjugation

Chemical modification enables the covalent attachment of ligands to exosome surfaces post-isolation using Click chemistry, EDC/NHS coupling and Biotin-streptavidin bridging (54). This method is helpful for ligands such as: Antibodies targeting viral surface glycoproteins (e.g., gp120 in HIV) and peptides that bind fungal or parasitic membrane antigens (55) Table 1.

##### 5.1.1.3 Lipid-Insertion Method

Ligands conjugated with lipids such as DSPE-PEG can be spontaneously incorporated into the lipid bilayer of exosomes, offering a non-genetic and modular strategy for surface functionalisation. For example, the DSPE-PEG conjugated to mannose or dectin-1 to target fungal β-glucans on *Cryptococcus* or *Candida* (56) Table 1.

As summarised in Table 1, tailoring exosome engineering requires close alignment with the infection biology of each pathogen. HIV-associated neuroinfections benefit from RVG- or anti-gp120-decorated exosomes for selective uptake by infected microglia (57). For Toxoplasma, aptamer-modified exosomes can target SAG1 antigens on parasitic cysts within neurons, enhancing delivery of gene-silencing tools. *Cryptococcus* infections, which exploit phagocytes to enter the CNS, are more effectively treated using mannose- or dectin-functionalised exosomes delivering antifungal drugs. HSV encephalitis, characterised by neuronal latency and reactivation, can be addressed through gD- or gB-binding ligand exosomes carrying antiviral siRNA. These pathogen-informed strategies highlight how exosome design must be customised to the distinct mechanisms of CNS pathogens rather than applying a uniform delivery approach (19, 58). Pathogen-specific ligand display offers several advantages, such as directing exosome-loaded drugs to infected cells, avoiding healthy tissues and increasing target specificity. It improved BBB navigation, such as ligands like RVG or transferrin can help exosomes cross the BBB and reach infected CNS regions—enhanced uptake by diseased cells, whereby ligands promote receptor-mediated endocytosis, boosting therapeutic payload internalisation (59). Also, effective synergistic therapy potential, for instance, targeting infected cells enables co-delivery of antimicrobials, siRNA, or immunomodulators precisely where needed (60, 61). On the other hand, there are limitations and challenges, including ligand immunogenicity, of which foreign peptides or antibodies may trigger immune reactions. Stability issues involving some ligands (e.g., aptamers) may degrade or detach under physiological conditions. Ligand density control, such as over- or under-expression of surface ligands, affects targeting efficiency and biodistribution. Heterogeneity of infected tissues, resulting from pathogen expression, may vary across stages or regions of infection, complicating uniform targeting (60, 62).

Our review study suggests future innovations focusing on dual-targeting ligands that combine BBB-penetrating ligands (e.g., T7 peptide) with pathogen-specific moieties for enhanced precision—designing ligands that activate or expose binding domains only under infection-specific conditions (e.g., pH-sensitive or enzyme-activated), like responsive ligands—using phage display, aptamer SELEX, or computational docking to discover novel ligands for emerging CNS pathogens (14, 63), such as ligand libraries and high-throughput screening. Pathogen-specific ligand display represents a transformative strategy in the field of exosome-based therapeutics (64). By tailoring the surface of exosomes with molecular tags that recognize and bind to infected brain cells, researchers can significantly enhance drug delivery platforms' accuracy, efficiency, and clinical potential. As techniques in ligand discovery, bioconjugation, and synthetic biology continue to evolve, pathogen-targeted exosome systems may soon become a mainstay in precision neuroinfectious disease treatment (65, 66).

#### 5.1.2 Implications of exosomes and BBB-targeting ligand display

Exosomes can be modified to express peptides that bind with endothelial receptors at the BBB, which improves CNS delivery. The rabies virus glycoprotein (RVG) peptide binds to nicotinic acetylcholine receptors, which helps it cross the BBB (67). Transferrin- or lactoferrin-conjugated exosomes use transferrin receptor-mediated endocytosis to enter the brain. Angiopep-2, TAT, and ApoE-derived peptides are promising ligands that help the CNS take up other substances. Genetic fusing of targeting moieties to exosomal membrane proteins like Lamp2b, CD63, or CD9 is the most common way to make these changes to the surface. This makes it possible for exosomes to show and express them stably (68, 69). Exosome therapy has to target the BBB so that therapeutic agents can reach the blood-brain barrier and enter infected brain areas. This method makes drug administration more effective while reducing off-target effects and systemic toxicity (70). These BBB-targeting ligands include receptor-mediated transcytosis (RMT) or adsorptive-mediated transcytosis, which help exosomes penetrate through endothelial cells. Enhanced accumulation of therapeutic exosomes in CNS compartments. Minimised off-target effects in peripheral organs. Increased therapeutic efficacy in neuroinfectious conditions such as HIV encephalitis, neurocryptococcosis, and cerebral toxoplasmosis (5, 71, 72) Table 2.

**Table 2:** Receptors at the BBB and their ligands.

Receptor at the BBB	Function	Common ligands used for targeting
Transferrin Receptor (TfR)	Iron transport is up-regulated in inflammation	Transferrin, T7 peptide, anti-TfR antibody
Low-Density Lipoprotein Receptor (LDLR)	Lipid uptake	ApoE, Angiopep-2 (73, 74)
Insulin Receptor (IR)	Glucose homeostasis	Insulin or IR-targeting peptides
Nicotinic Acetylcholine Receptor (nAChR)	Neurotransmission	RVG (rabies virus glycoprotein) peptide (75)
Leptin Receptor	Energy regulation	Leptin fragments
Scavenger Receptors (e.g., SR-B1)	Lipid and pathogen uptake	HDL-mimetic peptides (76)

#### 5.1.3 Prominent ligand display used in exosome engineering

##### 5.1.3.1 RVG peptide

The rabies virus glycoprotein (RVG) peptide has an affinity for nAChRs, which are expressed on neurons and BBB endothelial cells (77). When fused to exosomal membrane proteins like Lamp2b, RVG guides exosomes across the BBB and enables targeting of neuronal cells. This has been widely applied in RNA-based therapies for brain infections and neuroinflammation (78), Table 3.

**Table 3:** The prominent ligands used in exosome engineering.

Disease	Ligand used	Therapeutic payload	Benefit
HIV Encephalitis	RVG	siRNA or antiretroviral	Selective suppression of viral replication in the brain (79).
Neurocryptococcosis	Angiopep-2 + Mannose	Amphotericin B	Enhanced antifungal concentration in CNS tissues (80).
Cerebral Toxoplasmosis	T7 + anti-SAG1 aptamer	CRISPR or siRNA	Precise targeting of *T. gondii* within neurons (81)
Tuberculous Meningitis	Transferrin	Rifampicin, mRNA	Improved drug transport across the BBB to infected macrophages (82)

##### 5.1.3.2 T7 peptide

T7 is a synthetic peptide that binds selectively to the transferrin receptor. It provides a smaller, more stable alternative to full-length transferrin (83). Its use in exosome engineering supports targeted delivery across the BBB with a lower risk of immunogenicity or competition with endogenous transferrin (84).

##### 5.1.3.3 Angiopep-2

Angiopep-2 is designed to interact with the LDL receptor-related protein-1 (LRP1), which is highly expressed on the BBB endothelium. It is particularly effective in facilitating the transport of small molecules and nanoparticles into the brain. Exosomes decorated with Angiopep-2 have shown promise in treating glioblastoma and CNS infections (85).

##### 5.1.3.4 Transferrin

As a natural ligand of TfR, transferrin has been used to decorate exosomes to enhance CNS penetration. However, its performance may be affected by high levels of endogenous transferrin in circulation, necessitating dosage optimisation or combination with synthetic peptides (86) (Figure 2).

##### 5.1.3.5 Apoe fragments

Derived from apolipoprotein E, these peptides target LDL receptors and are particularly useful for delivering lipid-based or anti-inflammatory agents to the brain. They offer a biologically inspired approach to mimic native BBB transport mechanisms (87).

#### 5.1.4 Applications ligand display in brain-infecting pathogen therapy

Exosomes modified with ligands that target the BBB have shown promise in directly sending anti-viral, antifungal, and antiparasitic medicines to the brain. This focused method makes treatments work better against illnesses, including HIV encephalitis, neurocryptococcosis, and cerebral toxoplasmosis, while lowering the risk of systemic side effects. Researchers have examined exosomes with BBB-targeting ligands in several neuroinfectious illnesses (78, 88).

Adding BBB-targeting ligands to exosomes is an important step forward in creating treatments for brain-infecting viruses. These ligands make exosomes more effective at treating diseases by helping them get past the brain's natural defences and deliver medicine exactly where it is required. As ligand design and delivery methods continue to improve, BBB-targeted exosomes will likely change how neuroinfectious diseases are treated (78, 89, 90).

### 5.2 Genetic engineering tools for therapeutic payloads

In addition to surface targeting, exosomes can be genetically engineered to encapsulate specific nucleic acid-based therapeutics that modulate gene expression in target cells.

#### 5.2.1 CRISPR-Cas9 system

Scientists have made exosomes containing CRISPR/Cas9 ribonucleoprotein complexes or Cas9 mRNA coupled with guide RNAs (gRNAs). This lets them edit genes in latent viral reservoirs, including cutting out the HIV provirus from host DNA. This method could lead to possible cures for long-term CNS illnesses (91, 92).

#### 5.2.2 Small interfering RNA (siRNA) and antisense oligonucleotides (ASOs)

siRNAs and ASOs can silence pathogen-specific genes or modulate host immune responses. For instance, siRNAs targeting HIV tat/rev or HSV ICP0 have demonstrated viral suppression. ASOs have been designed to inhibit Cryptococcus virulence genes or T. gondii metabolic pathways (93, 94).

#### 5.2.3 Endosomal sorting and cargo loading

To enhance intracellular loading of therapeutic cargo into exosomes, donor cells are engineered with exosomal sorting motifs: Fusion proteins such as Lamp2b-GFP, CD63-Rab27a, or ALIX-binding domains are used to direct siRNAs, proteins, or CRISPR components into exosomes. MS2 bacteriophage coat protein-RNA aptamer systems have also been used to enrich RNA cargos selectively (95, 96).

### 5.3 Chemical engineering to enhance exosome stability and specificity

Beyond biological modification, chemical strategies have been widely applied to improve exosome robustness, payload stability, and cellular targeting (57). These includes;

#### 5.3.1 Click chemistry

Click chemistry is a chemical reaction that happens quickly, selectively, and without affecting biological molecules. It “clicks” molecular parts together in mild conditions. K. Barry Sharpless first came up with this idea in 2001, and it has changed the way bioconjugation is done, especially in drug delivery, nanomedicine, and diagnostics (97). Click chemistry makes it possible to change the surface of exosomes precisely and effectively add targeting ligands, imaging agents, or therapeutic payloads without damaging their structure or biological activity (98, 99). This is especially important for treatments that target pathogens that invade the brain, since they need to be delivered precisely to infected cells and be able to penetrate the blood-brain barrier (BBB). Click chemistry makes it easier to create new exosome-based drugs that are specifically designed to treat CNS infections (100).

The Copper (I)-catalysed azide-alkyne cycloaddition (CuAAC) is the most common click reaction. It makes a stable 1,2,3-triazole ring between an azide and an alkyne molecule. Click chemistry is known for having high specificity and yield, mild reaction conditions (physiological pH and temperature), bio-orthogonality (it does not interfere with biological processes), and few byproducts. In biological systems, copper-free “click” reactions like strain-promoted alkyne-azide cycloaddition (SPAAC) and inverse electron-demand Diels-Alder (IEDDA) reactions are preferred since they do not cause copper ions to be poisonous to cells (101, 102).

#### 5.3.2 Surface Functionalisation

Click chemistry enables covalent attachment of targeting ligands (e.g., antibodies, peptides, aptamers) to guide exosomes to pathogen-infected cells, BBB-penetrating moieties such as transferrin, lactoferrin, or RVG peptides for enhanced brain uptake and fluorescent or radiolabels for tracking and imaging *in vivo*. Some studies indicate that the exosome surface is first functionalised with azide groups, then a DBCO-modified RVG peptide is “clicked” on via SPAAC, allowing specific targeting to neurons infected with viral pathogens (103–105).

#### 5.3.3 Cargo Loading Enhancement

Click chemistry allows for internal or membrane-bound anchoring of therapeutic molecules: CRISPR-Cas9 components, siRNA or antisense oligonucleotides, Antimicrobial peptides and Anti-inflammatory small molecules. Such modifications ensure controlled orientation and stability, which are crucial for precise delivery in the CNS microenvironment (57).

### 5.4 Relevance of chemical engineering exosome in brain-infecting pathogen therapy

Click chemistry enhances the therapeutic utility of exosomes in several pathogen-targeted applications HIV-Associated Neuroinfections: Enables display of gp120-binding aptamers on exosomes for targeting infected microglia (106). Herpes Simplex Virus (HSV): Facilitates loading of anti-HSV siRNA inside exosomes with modified membrane proteins for enhanced neuronal uptake (57).Toxoplasmosis: Allows tethering of parasite-targeting antibodies on exosomal surfaces to direct them toward infected astrocytes (107). Tuberculous Meningitis (TBM): Supports BBB-targeted delivery of anti-TNF-α siRNA using click-functionalised exosomes (104, 105). The challenges for consideration include toxicity. A study showed copper toxicity (CuAAC) reaction that requires careful post-reaction purification or copper-free variants (93, 94). Ensuring batch-to-batch consistency in functionalisation is necessary for clinical-grade applications for scalability (108). Modifications must preserve exosome structure and prevent immune clearance (109), thereby enhancing the biocompatibility of ligands. Surface modifications may not always guarantee internalisation into the intended subcellular compartment (102), improving intracellular targeting. However, prospects could involve developing multifunctional click-compatible ligands (e.g., dual BBB-targeting and infection-targeting)—integration with bioorthogonal imaging for real-time tracking of therapeutic delivery (108). Scalable GMP-compliant click chemistry platforms for clinical-grade exosome production—application of click-to-release systems where cargo is activated only in the infected microenvironment (110). The click chemistry gives us a strong and flexible set of tools for changing the surfaces and interiors of exosomes. This makes it possible to deliver drugs to infected areas in the brain very selectively, efficiently, and stably. Exosomes are a critical molecular method for developing precision treatment for brain-infecting diseases because they work well with biological systems and have natural targeting and transport capabilities (110).

### 5.5 Conjugation strategies and chemistry

Aptamers can be conjugated to exosomes using several chemical and genetic methods:

#### 5.5.1 Covalent conjugation

Click Chemistry: Copper-catalysed azide-alkyne cycloaddition (CuAAC) enables site-specific attachment of alkyne-modified aptamers to azide-labelled exosomal surface proteins or vice versa.

Maleimide-thiol linkage: Aptamers modified with thiol groups can be linked to maleimide-functionalised lipids embedded in exosome membranes (55).

#### 5.5.2 Lipid insertion

Post-isolation, cholesterol or other lipid-conjugated aptamers are passively inserted into the lipid bilayer of exosomes via hydrophobic interaction, preserving aptamer functionality and exosome integrity (111).

#### 5.5.3 Genetic engineering

Parent cells can be transfected to express aptamer-binding domains fused with exosomal membrane proteins (e.g., Lamp2b, CD63), allowing selective display of aptamers on exosomal surfaces after loading (45).

### 5.6 Aptamer conjugation in exosome engineering for targeted CNS infection therapy

Aptamer conjugation has become a flexible and valuable way to make exosomes functional for targeted delivery, notably in the problematic area of central nervous system (CNS) infections (112). The Systematic Evolution of Ligands by Exponential Enrichment (SELEX) technique chooses aptamers, which are short, single-stranded DNA or RNA oligonucleotides, to bind strongly and selectively to a wide range of targets, such as proteins, cells, and tiny molecules (45, 113). The rationale for Aptamer Use in brain-targeted exosome therapy is that aptamers can resemble antibodies in recognising targets because of their unique structural folding. They also have benefits such as being less immunogenic and poisonous. Easier to make and change, better stability in various physiological circumstances and a high binding selectivity with an affinity of nanomolar to picomolar (114). In the context of CNS-targeted therapies, aptamers can be selected to bind to: Blood-brain barrier (BBB) transporters (e.g., transferrin receptor, insulin receptor), Pathogen-specific antigens (e.g., surface proteins of Neisseria meningitidis, Cryptococcus neoformans, or viral envelope proteins), infected cells or inflammatory microenvironment markers (e.g., ICAM-1, VCAM-1, and CD44) (114, 115).

#### 5.6.1 Evidence applications of aptamer-functionalised in CNS infection targeting

##### 5.6.1.1 BBB translocation

Aptamers that bind transferrin or insulin receptors let exosomes penetrate the BBB through receptor-mediated transcytosis. For example, exosomes with transferrin receptor aptamer (like TVRA) attached to them and filled with siRNA or antibiotics worked better at getting into the central nervous system in models of bacterial meningitis (74).

##### 5.6.1.2 Pathogen-Directed targeting

Exosomes containing aptamers that target pathogen virulence factors (such as HIV gp120 and Listeriolysin O) can carry anti-viral or anti-bacterial drugs directly to infected cells, reducing systemic exposure risk (116).

##### 5.6.1.1 Neuroinflammation modulation

Aptamers that bind to markers of neuroinflammation, such as TNF-α, IL-6 receptors, and activated microglia markers, can send exosomes with anti-inflammatory miRNAs or medicines to sick parts of the brain (117, 118). However, aptamers are better than other targeting ligands like antibodies and peptides because they are easier to make and change, cheaper, and penetrate better. After all, they are smaller and have a lower risk of activating the immune system, which is important for repeated CNS therapeutic dosing (89, 90, 119).

Furthermore, the challenges are that nucleases may degrade aptamers; however, chemical modifications (e.g., 2′-fluoro, 2′-O-methyl substitutions) can enhance nuclease resistance. Scale-up of aptamer-exosome conjugates under GMP conditions remains underdeveloped, leading to manufacturing complexity. High mutation rates in pathogens may necessitate re-selection of aptamers due to target variability (120).

Future research engagement will be to develop multiplexed exosomes conjugated with multiple aptamers for simultaneous targeting of co-infections or complex CNS environments. Integration of aptamer-exosome systems with stimuli-responsive release mechanisms. Clinical-grade aptamer libraries for broader CNS pathogen spectrum coverage (120). Aptamer conjugation will make exosome-based delivery platforms more accurate for treating CNS infections. This technique has much potential for turning molecular discoveries into next-generation, tailored drugs for brain-infecting pathogens thanks to improvements in aptamer selection, conjugation chemistry, and exosome bioengineering (121).

## 6 Lipid modification in exosome engineering for targeted CNS infection therapy

**Figure 3:**
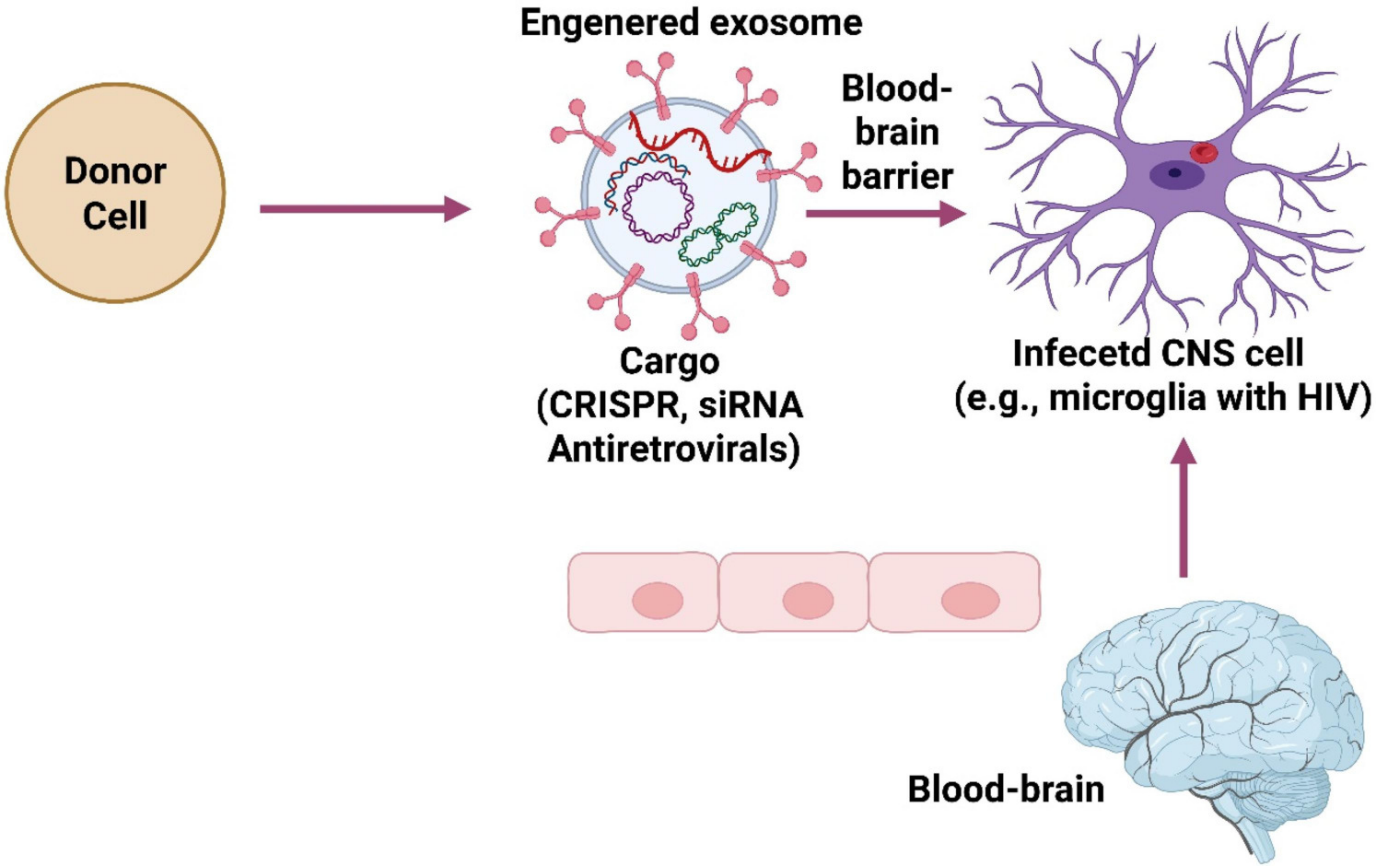
Exosome-based targeted therapy for brain infections.

Changing the lipids on exosomes is a key part of exosome engineering since it allows the change of the surface of exosomes to make them better at targeting, staying stable, and delivering their cargo. Exosomes are naturally lipid-bilayer vesicles; thus, changing their lipid parts is a safe and effective way to deliver drugs better, especially for hard-to-reach areas like the brain, where they can target infectious agents (45) (Figure 3). The rationale for lipid modification in CNS therapeutics is that exosomes have a lipid bilayer comprising cholesterol, sphingomyelin, phosphatidylserine, and other phospholipids similar to cell membranes. These structural commonalities give targeted delivery and intracellular trafficking significant benefits (115). However, native exosomes do not naturally go to infected brain areas. Changing lipids can help get beyond the BBB, avoid immune clearance, and stay longer at infected or inflamed areas. Changing lipids makes exosomes more stable in the blood, which helps them penetrate through the blood-brain barrier (BBB) and adhere to diseased or inflamed brain tissues more easily. It also helps ligands (including aptamers, peptides, and antibodies) stick to the surface of the exosome (45).

### 6.1 Strategies for lipid modification of exosomes

Several methods have been developed to alter or enhance the lipid composition of exosomes either pre-isolation (during biogenesis) or post-isolation (after purification). The post-isolation lipid insertion strategy involves passively inserting or incorporating modified lipids into the exosome membrane (45). For instances, cholesterol-conjugated ligands such as molecules like miRNA, aptamers, or peptides can be attached to cholesterol and inserted into exosome membranes via hydrophobic interactions (73, 74). Also, PEGylated lipids (e.g., DSPE-PEG), here the insertion of polyethene glycol (PEG)-linked lipids can prolong circulation time, reduce opsonisation and clearance by macrophages, and provide anchor points for further ligand attachment (e.g., via NHS ester or maleimide groups) (122). Lastly is targeting ligand-lipid conjugates such as RVG (rabies virus glycoprotein peptide), transferrin, or folate can be conjugated to lipids like DSPE and inserted to enhance brain or pathogen-specific targeting (123). On the other hand, is the pre-isolation lipid engineering in donor cells that involves lipid supplementation in culture, were the parent cells are cultured with exogenous modified lipids, which get incorporated into budding exosomes (124). Also, genetic modification of donor cells that involves overexpression of enzymes like ceramide synthase or phospholipid remodelling enzymes changes the lipid profile of secreted exosomes, improving membrane fluidity or targeting properties (125).

### 6.2 Applications of lipid modified exosomes in targeted therapy of brain-infecting pathogens

Lipid-modified exosomes have been explored in multiple therapeutic contexts, such as:

#### 6.2.1 BBB penetration enhancement

RVG–lipid modified exosomes have shown the ability to deliver siRNA or drugs across the BBB by targeting the nicotinic acetylcholine receptor. Lipid-conjugated PEGylation delays RES (reticuloendothelial system) clearance and promotes accumulation in brain tissue (126).

#### 6.2.2 Pathogen- or inflammation-targeted delivery

Modified lipids attached to mannose or ICAM-1 targeting ligands can send exosomes to activate the immune system or infected endothelial cells. Phagocytic cells (like microglia) are more likely to take up exosomes modified with phosphatidylserine analogues. This makes them suitable for delivering drugs directly to the site of bacterial meningitis or neuroinvasive fungal infections (127–129).

#### 6.2.3 Drug loading enhancement

Lipid-modified exosomes can more efficiently encapsulate hydrophobic anti-pathogen drugs (e.g., amphotericin B, rifampicin, acyclovir). Thermosensitive lipids allow triggered cargo release in response to localised heating or inflammation (44, 127–129).

Among the limitations and challenges of exosomes' applications in targeted therapy of brain-infecting pathogens are membrane integrity-associated excessive modification, which may disrupt membrane fluidity or exosome uptake efficiency. Inconsistent lipid insertion or orientation can affect targeting and therapeutic outcomes. Specific synthetic lipid moieties may elicit immune responses *in vivo*. Reproducibility and cost-efficiency in clinical-grade lipid modifications are still under optimisation (109, 130). However, future research perspectives could focus on stimuli-responsive lipids (e.g., pH-sensitive, redox-sensitive, thermosensitive) for controlled drug release in infected CNS regions. Exosomes with lipid modifications can do more than one thing, like imaging agents, targeting ligands, and therapeutic payloads for theranostic uses. Synthetic exosome mimetics that use specific lipid compositions to copy the qualities of natural exosomes while making them easier to manage. Changing the lipids in exosomes is a game-changing way to make medicines for CNS illnesses. Exosomes can be made better for targeted delivery, longer circulation, and efficient payload release by changing the structure of the lipids. New discoveries in lipid chemistry, membrane biology, and nanomedicine will improve these tactics, giving doctors more options for treating deadly brain infections with precision and little harm to the rest of the body (131, 132).

## 7 Exosome-based drug delivery systems against brain pathogens

Exosome-based drugs are a game-changer for treating central nervous system infections (CNS) infections, especially when traditional treatments don't work well because they don't get through the blood-brain barrier (BBB), are toxic, or aren't targeted enough (16). Exosomes provide a flexible, biocompatible, and targeted way to distribute a wide range of drugs, from small compounds and biologics to gene-editing systems, straight to brain areas that are diseased or inflamed (133). Here, we talk about new improvements in exosome-mediated delivery systems that are made to treat viral, fungal, protozoal, and neuroinflammatory disorders that affect the brain.

### 7.1 Antiretroviral delivery for HIV-associated CNS infections

Conventional antiretroviral therapy (ART) is often ineffective in eliminating HIV reservoirs in the brain due to limited drug penetration and persistence of the virus in microglial cells. Engineered exosomes have shown promise in overcoming these barriers (134).

#### 7.1.1 Tenofovir and efavirenz delivery

Exosomes from macrophages or mesenchymal stem cells have wrapped up tenofovir and efavirenz and sent them straight to the CNS. These exosomes show that they can cross the BBB more easily, deliver drugs directly to infected microglia, and release them over time, which lowers systemic toxicity and raises CNS drug concentrations (135).

#### 7.1.2 CRISPR-Based HIV gene editing tools

Exosomes could transport CRISPR-Cas9 ribonucleoproteins, mRNA, and guide RNAs targeting integrated HIV DNA (e.g., LTR, gag, or tat/rev regions). Based on exosomes, these methods provide a non-viral, immunologically safer way to cut out genes or stop the production of proviral HIV in CNS reservoirs, which could lead to functional cure treatments.

### 7.2 Antifungal strategies against cryptococcus neoformans

Current antifungal drugs have toxic profiles and don't get into the brain very well, which makes it very hard to treat cryptococcal meningitis. Researchers are working on exosome-based delivery systems to make them more effective and less harmful (136).

#### 7.2.1 Amphotericin B-loaded exosomes

Amphotericin B is very toxic when given systemically, so it benefits from being put in exosomes, which makes it more soluble, targets the brain better, and lowers its toxicity to the kidneys. Preclinical models have shown reduced fungal burden and improved survival using exosome-encapsulated formulations (137).

#### 7.2.2 Fluconazole-loaded exosomes

Exosomes functionalised with mannose ligands have been used to selectively target Cryptococcus cells by binding to fungal surface mannoproteins, thereby enhancing the antifungal action of fluconazole while reducing off-target effects (138, 139).

### 7.3 Antiprotozoal applications for toxoplasma gondii

Toxoplasmic encephalitis remains a significant cause of mortality in immunocompromised populations, and new molecular approaches are needed to overcome the parasite's ability to form latent cysts in the brain (140).

#### 7.3.1 SiRNA-loaded exosomes

Scientists have made exosomes that deliver siRNAs that target important virulence genes of T. gondii, such as ROP18 (rhoptry protein 18), which helps the parasite avoid the immune system, and SAG1 (surface antigen 1), which helps the parasite invade host cells. In preclinical animals, these siRNA-loaded exosomes, especially when paired with ligands that can cross the blood-brain barrier (such as RVG or transferrin), have been shown to stop parasite reproduction, lower cyst burden, and enhance survival (139, 141).

### 7.4 Implication of exosomes in neuroprotective payloads to counter CNS damage

Infections in the CNS can cause neuroinflammation, oxidative stress, and damage to neurons, long after the pathogens are gone. Exosomes can also transport neurotropic and anti-inflammatory drugs to help rehabilitate and stop long-term neurological problems (142).

#### 7.4.1 Brain-derived neurotrophic factor (BDNF) and glial cell line-derived neurotrophic factor (GDNF)

When given through exosomes, these neurotrophins have been demonstrated to help neurons stay alive, make synapses more flexible, and fix broken brain circuits in models of viral encephalitis and HIV-related neurodegeneration (143).

#### 7.4.2 MiRNA-Loaded exosomes

Exosomes high in anti-inflammatory miRNAs like miR-124, miR-146a, or miR-21 can change how microglia work, stop pro-inflammatory cytokines (such as TNF-α and IL-6), and help neuroinflammation go away. These miRNA therapeutics help pathogen-specific treatments by reducing harm to the immune system in the CNS (144). Exosome-based delivery systems provide a multi-faceted way to treat CNS infections by carrying anti-bacterial and gene-editing medicines and neuroprotective and anti-inflammatory compounds. Exosomes are one of the most promising platforms for next-generation treatments for brain-infecting diseases because they can transport different payloads, target specific cell types, and penetrate biological barriers. Continued progress in standardisation, biodistribution studies, and clinical-grade manufacturing will be essential to translate these innovations into human therapies (145).

## 8 Implications of exosomes on routes of administration and bioavailability

The therapeutic efficacy of exosome-based delivery systems in treating CNS infections is highly dependent on the route of administration and the biodistribution of these nanocarriers (146). Given the complexity of the blood-brain barrier (BBB) and latent pathogen reservoirs in specialised CNS compartments, optimising how exosomes are delivered—and understanding where they localise—is essential for clinical success (147).

### 8.1 Delivery routes for CNS-targeted exosomes

Several administration routes have been explored to maximise exosome delivery to the brain. The choice of route influences distribution, onset of action, and clinical feasibility (148).

#### 8.1.1 Intranasal delivery

A non-invasive and patient-friendly route that enables exosomes to bypass the BBB via the olfactory and trigeminal nerve pathways. The various studies show that RVG-tagged exosomes delivered intranasal accumulations in the olfactory bulb, hippocampus, and cortex within hours and are particularly effective for siRNA and CRISPR-Cas9 cargo in models of viral encephalitis and neuroHIV (149).

#### 8.1.2 Systemic (intravenous) administration

While systemic injection (IV) is the most common method, the reticuloendothelial system may rapidly clear unmodified exosomes. Surface modification with ligands like transferrin, RVG, or lactoferrin enhances BBB crossing and CNS uptake. Bio distribution studies confirm CNS accumulation, especially in inflammatory or infected brain regions, due to enhanced permeability (150).

#### 8.1.3 Intrathecal injection

Direct administration into the cerebrospinal fluid (CSF) spreads the drug throughout the CNS and avoids systemic metabolism. Exosomes can reach the spinal cord, meninges, and deep brain regions when injected into the spine. They are employed in models of fungal meningitis and toxoplasmic encephalitis (127–129).

#### 8.1.4 Intraventricular injection

It provides high local concentrations of exosomes in the brain's ventricular system and is used for preclinical studies targeting deep-seated infections or delivering neurotropic factors. Invasive but highly effective in rodents for testing exosome pharmacokinetics and payload release (151).

### 8.2 Imaging and tracking of exosomes

Exosomes must be tracked *in vivo* to evaluate distribution, clearance, and CNS specificity to assess their therapeutic relevance. Multiple labelling and imaging techniques have been developed, including;

#### 8.2.1 Radiolabeling

Isotopes like 99mTc, 111In, and 64Cu are conjugated to SPECT or PET imaging exosomes, providing quantitative biodistribution data. This enables longitudinal tracking in small animals and non-human primates and demonstrates preferential accumulation in the brain, spleen, liver, and infected sites (152).

#### 8.2.2 Fluorescence imaging

Lipophilic dyes such as DiR, DiI, and PKH26 label exosomal membranes. This will allow real-time tracking in live animal imaging systems (IVIS) and histological studies, and also confirms accumulation in microglia, astrocytes, and infected neuronal zones post-administration (153, 154).

#### 8.2.3 MRI contrast agents

Incorporation of superparamagnetic iron oxide nanoparticles (SPIONs) into exosomes permits visualisation by MRI. This offers high-resolution anatomical mapping of exosome localisation in brain tissues (155).

### 8.3 Evidence of CNS accumulation and specificity

Several preclinical studies have confirmed the ability of engineered exosomes to cross the BBB and selectively target CNS pathogens. RVG-modified exosomes delivering anti-HIV siRNA showed specific uptake by HIV-infected microglia and reduced viral replication in the CNS. In Cryptococcus infection models, mannose-modified exosomes loaded with amphotericin B localised preferentially to infected meninges and reduced fungal burden significantly. Exosomes tagged with T. gondii-specific aptamers demonstrated high specificity for parasitic cysts in the brain and suppressed cystogenesis when carrying gene-silencing cargo (156). This review again shows that optimising delivery routes and surface engineering are important for getting targeted therapy, reducing off-target effects, and increasing the therapeutic index in CNS infections. By carefully choosing the right ways to give exosomes and doing precise molecular engineering, we may get them to the CNS areas more effectively. Modern tracking technology gives us much information about how exosomes move through the body and where they go. These new ideas make using exosome-based medicines in clinical settings possible to treat hard-to-treat brain infections (157).

## 9 Translational and clinical considerations

Exosome-based therapies show great potential for CNS infections; however, their clinical application faces substantial technical, regulatory, and manufacturing impacts. Despite preclinical progress, there are no approved therapies due to a number of issues, including logistical challenges with bulk production, safety verification, regulatory issues, and undefined quality control measures (8).

### 9.1 Scalability and manufacturing challenges

Exosome production remains difficult to scale reproducibly, with yields varying by donor cell type, culture conditions, and passage number. Although bioreactors and tangential flow filtration (TFF) improve yield and purity compared to ultracentrifugation or precipitation, batch-to-batch reproducibility and GMP-compliant release criteria (identity, purity, potency) are not yet standardized (180).

### 9.2 Immunogenicity and safety profiles

While generally considered biocompatible, engineered or allogeneic exosomes may carry PAMPs or inflammatory molecules that raise immunogenicity concerns (181). PEGylation and membrane engineering (e.g., CD47 expression) can mitigate risk, but regulators require extensive long-term toxicology, biodistribution, and off-target data. Preclinical reports of low toxicity for exosome-based siRNA, CRISPR, or antifungal delivery (158) remain too limited in scale to satisfy clinical standards (159).

### 9.3 Regulatory landscape and clinical trials

Exosome therapies fall into a gray zone between biologics, cell-derived products, and nanomedicine, complicating approval pathways. Regulatory agencies (FDA, EMA, CDSCO) demand source traceability, sterility, reproducible characterization, and validated potency assays. Although early-phase trials in cancer and neurodegeneration demonstrate safety and CNS targeting, no product has yet been approved for CNS infections, reflecting the regulatory bottlenecks (160).

### 9.4 GMP compliance and quality control

Currently, no harmonized GMP standards exist for exosome therapeutics. Key analytical markers—particle size (30–150 nm), zeta potential, protein cargo (CD9, CD63, CD81), and RNA content—are inconsistently applied, and robust potency assays are undeveloped. Lack of clear release specifications and validated QC assays for clinical-grade exosomes remains a critical barrier to regulatory approval (161). Also, the challenge of batch-to-batch consistency requires stringent protocols for donor cell characterisation, culture conditions, and post-processing storage (e.g., lyophilisation, cryopreservation). Like Quality Control Technologies are Nanoparticle tracking analysis (NTA), Western blotting and flow cytometry for surface markers (CD63, CD81, CD9) and High-throughput RNA sequencing for transcript cargo profiling (162).

### 9.5 Toward personalised medicine and point-of-care delivery

Exosome platforms hold significant promise for individualised therapies, especially in settings where rapid, tailored interventions are needed:

#### 9.5.1 Personalised drug delivery

Exosomes from patients could contain gene-editing tools or targeting ligands that are distinct to each patient. This would lower immune responses and make the treatment more effective. This method could help get rid of latent reservoirs with great accuracy in CNS illnesses like neuroHIV or toxoplasmic encephalitis.

#### 9.5.2 Point-of-care potential

Modular bioreactors and lab-on-chip separation devices could be used to make portable production and management systems that can be used on-site. This is especially important in areas with few resources or many disease outbreaks, where treatments need to be given quickly. The journey from bench to bedside for exosome-based therapies targeting brain infections is promising but complex. Addressing scalability, immunogenicity, GMP compliance, and regulatory alignment is essential to unlock the full therapeutic potential of this platform. Advances in standardisation, automation, and personalised approaches will accelerate clinical translation and open new avenues for treating challenging CNS infections with precision and safety (163).

## 10 Limitations of exosome-based delivery

Exosome-based delivery platforms have many technical challenges that prevent clinical translation. One of the biggest challenges is batch-to-batch variability, since yield, cargo composition, and functional activity are dependent on cell source, culture conditions, and scale-up process, making it difficult to get reproducible results. In addition, exosome preparations may be contaminated with other forms of extracellular vesicles (microvesicles, apoptotic bodies) and non-vesicular biomolecules, such as protein aggregates, lipoproteins, and RNA–protein complexes, making functional assessment complicated and figuring out dosage issues difficult and raising safety concerns (164). Additionally, there is a lack of standardized isolation and purification methods. The most routinely used approaches for isolating and purifying exosomes include ultracentrifugation, precipitation, size-exclusion chromatography, and tangential flow filtration. Each of these methods yields different amounts of exosomes with differing levels of purity and different scalability, and no agreement on how to create clinical-grade protocols (165). Lastly, quality control and potency assays are underdeveloped, and the commonly used exosome markers (CD9, CD63, CD81) reveal identity, but do not measure therapeutic efficacy. These issues—variability, contamination, non-standardized purification, and potency assays—are all significant barriers that need to be overcome if we are to translate exosome therapeutics into clinical use (109).

## 11 Future perspectives and research directions

The area of medication delivery using exosomes is moving quickly forward and can potentially change how we diagnose and treat diseases. However, a few important areas still need more research to completely understand what exosomes can do, especially in complicated illnesses, including the central nervous system (CNS), cancer, and infectious disorders. We must also plan how exosome research and clinical translation will go over the next ten years (51, 166).

### 11.1 Mechanistic insights into exosome–pathogen interactions

One critical area of future research involves elucidating the molecular and cellular mechanisms underlying exosome-pathogen interactions. Exosomes can inhibit or facilitate pathogen dissemination by serving as decoys or carriers. Understanding: How viruses like HIV, SARS-CoV-2, or Zika exploit exosomal pathways, the role of exosomal cargo (miRNAs, proteins, lipids) in modulating immune responses, Host-pathogen communication through exosomal signaling, will not only improve our comprehension of disease progression but may also enable the repurposing of exosomes as anti-viral or antimicrobial delivery tools. High-resolution proteomics and single-exosome RNA sequencing technologies will be instrumental in uncovering these mechanisms (166).

### 11.2 Synthetic exosomes and hybrid delivery systems

Natural exosomes are safe for living things, but they have problems with scalability, heterogeneity, and loading cargo efficiently. So, people have been paying attention to the rise of synthetic exosomes (or biomimetic vesicles). These manufactured structures copy the physical and biological properties of natural exosomes, which allow changes in their composition and surface in a regulated way, encapsulate drugs more effectively and precisely, and make them more stable and last longer. Hybrid technologies, including exosome-liposome hybrids or exosome-coated nanoparticles, also have the advantage of targeting naturally and having engineered functionality. These platforms may prove especially useful in crossing the blood-brain barrier (BBB) or targeting hard-to-reach tissues (167).

### 11.3 Integration with AI-driven exosome design, Bio printing, and organ-on-chip models

The integration of computational biology and Artificial intelligence (AI) is being increasingly used in exosome engineering, including cargo loading, surface modification, targeting, and quality control. Machine learning can help predict the most efficient encapsulation of RNA or drug; while there are programs (e.g., with AlphaFold) that could assist with designing ligands or peptides for improved targeting toward the brain (168). For example, AI-guided optimization of miRNA loading for glioblastoma treatment, as well also computational docking, were used to support engineering RVG-tagged exosomes that can cross the blood–brain barrier (169). AI has also made patient-specific predictions by analyzing exosomal biomarkers and supporting personalized medicine. Next generation, AI-driven imaging is expected to improve reproducibility and the quality of evidence that goes into manufacturing. The incorporation of robotics for liquid handling and integrating AI with high-throughput screening and design may help lead to more efficient scaled-up exosome-based therapies for clinical use (170). Moreover, 3D bio printing can aid in fabricating exosome-loaded scaffolds for regenerative applications, such as neural repair or cardiac tissue engineering. Alongside this, organ-on-chip platforms (like brain-on-chip or liver-on-chip) are being developed to mimic *in vivo* environments, providing a more accurate preclinical assessment of exosome-based therapeutics, including toxicity and permeability studies (171).

### 11.4 Combinatorial therapeutic approaches

Combinatory Medicines Combinatory medicines refer to therapies that include two or more agents that work synergistically, have better resistance profiles, or are less toxic than monotherapies. This approach is increasingly important in multifactorial diseases including cancer, infections, and neurological disorders with multiple therapeutic targets (172). For instance, chemotherapy plus immunotherapy (e.g., PD-1 inhibitor plus paclitaxel) has demonstrated improved survival rates in patients with non-small cell lung cancer (173). In infectious diseases, antiretroviral therapy (ART) involves combination therapies that target different stages of the HIV life cycle to reduce viral resistance. The use of a multiple-drug regimen to treat tuberculosis (isoniazid, rifampicin, ethambutol, pyrazinamide) is essential to ensure resistant strains do not develop and to ensure that patients ultimately receive a curative treatment (174). In the neurological context, levo-dopa with carbidopa used as a co-medication in Parkinson's disease (PD) to increase efficacy and decrease undesirable systemic side effects. There is ongoing research into co-delivery of drugs (e.g., doxorubicin with siRNA) via various delivery vehicles including nanoparticles or exosomes to improve delivery targeting and maximize therapeutic outcomes (24, 25, 175).

### 11.5 Standardisation, regulatory advancements, and clinical translation

While the exosome engineering for targeted therapy for brain-infecting pathogens is promising, the clinical application of exosomes still requires standardised isolation, characterisation, and storage protocols (176). Therefore, future research could focus on creating potency tests and quality control measures for clinical-grade exosomes, setting up production lines that follow good manufacturing practices (GMP), and making rules for making synthetic or hybrid exosome compositions. Long-term clinical trials are also necessary to determine the long-term safety, immunogenicity, and therapeutic results of exosome-based therapeutics in different patient groups. Exosome research is at a crucial point, with progress in several fields coming together to realise their therapeutic potential fully (158). Future work should harness molecular tools, delivery approaches, and translational challenges, using synthetic biology, nanotechnology and AI to enhance targeting, scalability, and safety for exosome based therapies for brain pathogens. As the area grows, it will be important to deal with mechanistic gaps, translational obstacles, and regulatory hurdles to successfully move from bench to bedside (177, 178).

## 12 Conclusion

Exosome engineering represents a paradigm shift in targeted drug delivery, particularly for challenging conditions like central nervous system (CNS) infections. These infections—including viral encephalitis, bacterial meningitis, fungal neuroinfections, and parasitic diseases—are often life-threatening and difficult to treat due to the presence of the blood–brain barrier (BBB), which limits the entry of most therapeutics into the brain. One of the most remarkable attributes of exosomes is their innate ability to traverse the BBB, a property not shared by many conventional drug delivery vehicles. This makes them uniquely equipped to deliver therapeutic agents directly to infected or inflamed brain tissues. Furthermore, exosomes can be engineered to carry customised cargo, such as: anti-viral or anti-bacterial drugs, Anti-inflammatory molecules, Small interfering RNA (siRNA), microRNA (miRNA), or mRNA, Proteins or peptides that modulate host immune responses. These capabilities allow exosomes to act as passive carriers and bioactive modulators of the host–pathogen interface. Their biocompatibility, low immunogenicity, and ability to mimic natural intercellular communication further enhance their potential as delivery platforms for CNS-targeted therapy. Additionally, exosomes can be derived from various cell types—such as dendritic cells, macrophages, mesenchymal stem cells, or even brain endothelial cells—to fine-tune their surface markers and targeting properties. When engineered with specific ligands or surface peptides, they can achieve cell-type-specific delivery, reducing off-target effects and increasing therapeutic precision. Despite these advantages, the field must overcome several translational challenges, including: Scalable and reproducible manufacturing, Comprehensive safety profiling, regulatory approval hurdles, and standardised characterisation protocols. Nevertheless, with continued progress in nanotechnology, synthetic biology, and systems pharmacology, integrating exosome-based therapies into clinical practice appears increasingly feasible. Exosome engineering holds tremendous promise for transforming the treatment landscape of CNS infections. By enabling precision-targeted delivery, promoting host-pathogen modulation, and facilitating cross-BBB drug transport, exosomes are poised to become next-generation therapeutic vectors. A key strength of exosome engineering lies in its adaptability to pathogen-specific challenges. By considering where pathogens reside (e.g., microglia, neurons, meninges), how they persist (latency, cyst formation, immune evasion), and which surface markers they express, exosome platforms can be rationally tailored to deliver precise therapeutic payloads. This pathogen-focused approach not only enhances therapeutic efficacy but also reduces off-target effects, positioning exosomes as uniquely suited for tackling diverse CNS infections. As interdisciplinary collaborations expand and mechanistic understanding deepens, exosome-based therapies could redefine how we approach and manage complex neuroinfectious diseases, potentially improving patient outcomes worldwide.
